# PuraStat in gastrointestinal bleeding: results of a prospective multicentre observational pilot study

**DOI:** 10.1007/s00464-021-08589-6

**Published:** 2021-06-15

**Authors:** Federica Branchi, Rolf Klingenberg-Noftz, Kristina Friedrich, Nataly Bürgel, Severin Daum, Juliane Buchkremer, Elena Sonnenberg, Michael Schumann, Christoph Treese, Hanno Tröger, Donata Lissner, Hans-Jörg Epple, Britta Siegmund, Andrea Stroux, Andreas Adler, Winfried Veltzke-Schlieker, Daniel Autenrieth, Silke Leonhardt, Andreas Fischer, Christian Jürgensen, Ulrich-Frank Pape, Bertram Wiedenmann, Oliver Möschler, Maximilian Schreiner, Mathias Z. Strowski, Volkmar Hempel, Yvonne Huber, Helmut Neumann, Christian Bojarski

**Affiliations:** 1 Campus Benjamin Franklin, Medizinische Klinik mit Schwerpunkt Gastroenterologie, Infektiologie und Rheumatologie, Charité—Universitätsmedizin Berlin, Hindenburgdamm 30, 12203 Berlin, Germany; 2 DRK-Krankenhaus Grevesmühlen, Gastroenterologie und Allgemeine Innere Medizin, Klützer Straße 13-15, 23936 Grevesmühlen, Germany; 3 Institut für Biometrie und Klinische Epidemiologie, Charité—Universitätsmedizin Berlin, Hindenburgdamm 30, 12200 Berlin, Germany; 4 Campus Virchow Klinikum, Medizinische Klinik mit Schwerpunkt Gastroenterologie und Hepatologie, Charité—Universitätsmedizin Berlin, Augustenburger Platz 1, 13353 Berlin, Germany; 5 Campus Mitte, Medizinische Klinik mit Schwerpunkt Gastroenterologie und Hepatologie, Virchowweg 10, 10117 Berlin, Germany; 6 Niels-Stensen-Kliniken, Marienhospital Osnabück GmbH, Bischhofstraße 1, 49074 Osnabrück, Germany; 7 Bundeswehrkrankenhaus Berlin, Klinik für Innere Medizin, Scharnhorststraße 13, 10115 Berlin, Germany; 8 Park-Klinik Weißensee, Gastroenterologie und Onkologie, Klinik für Innere Medizin, Schönstr. 80, 13086 Berlin, Germany; 9 Klinikum Mittleres Erzgebirge gGmbH, Klinik für Innere Medizin, Alte Marienberger Straße 52, 09405 Zschopau, Germany; 10 I. Medizinische Klinik Und Poliklinik, Universitätsmedizin der Johannes Gutenberg-Universität Mainz, Langenbeckstr. 1, 55131 Mainz, Germany

**Keywords:** PuraStat, Haemostasis, Gastrointestinal bleeding

## Abstract

**Background:**

A recently developed haemostatic peptide gel for endoscopic application has been introduced to improve the management of gastrointestinal bleeding. The aim of this pilot study was to evaluate the feasibility, safety, efficacy and indication profiles of PuraStat in a clinical setting.

**Methods:**

In this prospective observational multicentre pilot study, patients with acute non-variceal gastrointestinal bleeding (upper and lower) were included. Primary and secondary application of PuraStat was evaluated. Haemoglobin, prothrombin time, platelets and transfusion behaviour were documented before and after haemostasis. The efficacy of PuraStat was assessed during the procedure, at 3 days and 1 week after application.

**Results:**

111 patients with acute gastrointestinal bleeding were recruited into the study. 70 percent (78/111) of the patients had upper gastrointestinal bleeding and 30% (33/111) had lower gastrointestinal bleeding. After primary application of PuraStat, initial haemostatic success was achieved in 94% of patients (74/79, 95% CI 88–99%), and in 75% of the patients when used as a secondary haemostatic product, following failure of established techniques (24/32, 95% CI 59–91%). The therapeutic success rates (absence of rebleeding) after 3 and 7 days were 91% and 87% after primary use, and 87% and 81% in all study patients. Overall rebleeding rate at 30 day follow-up was 16% (18/111). In the 5 patients who finally required surgery (4.5%), PuraStat allowed temporary haemostasis and stabilisation.

**Conclusions:**

PuraStat expanded the therapeutic toolbox available for an effective treatment of gastrointestinal bleeding sources. It could be safely applied and administered without complications as a primary or secondary therapy. PuraStat may additionally serve as a bridge to surgery in order to achieve temporary haemostasis in case of refractory severe bleeding, possibly playing a role in preventing immediate emergency surgery.

**Supplementary Information:**

The online version contains supplementary material available at 10.1007/s00464-021-08589-6.

The increase in incidence of upper gastrointestinal bleeding, especially in elderly patients or those with comorbidities, has major implications for future healthcare systems [1]. The associated mortality of 10% remained constant over the past two decades [2]. The most common cause of acute non-variceal upper gastrointestinal bleeding is peptic ulcer disease, accounting for 25–67% of all cases [3]. Treatment of non-variceal gastrointestinal bleeding is challenging, in particular in tumour bleeding or when established techniques such as injection therapy, hemoclips or thermocoagulation fail.

Within recent years haemostatic powders (EndoClot, Hemospray, Nexpowder) have become common tools for managing diffuse bleeding or bleeding localised in difficult anatomical regions. To facilitate their application either an air compressor (EndoClot) or a carbon dioxide cartridge (Hemospray) are required. Studies have shown both powders are considered to be equally effective [4, 5]. However, the clinical use of these powders may, on occasion be hampered by clogging of the application catheter, which may occur when the powder is in contact with fluid inside the catheter. Additionally, visibility of the bleeding site and landmarks can become obscured following application of the powders owing to their opaque nature.

Another haemostatic agent for use in endoscopic therapy has recently been introduced, a biocompatible synthetic peptide gel, PuraStat® (3D Matrix Europe SAS, Caluire-et-Cuire, France). PuraStat is a viscous transparent gel, which utilises the self-assembling peptide technology, and is indicated for haemostasis of oozing bleeding in parenchyma of solid organs, vascular anastomoses and small blood vessels or capillaries of the GI tract. It is additionally indicated for reduction of delayed bleeding following colonic endoscopic submucosal dissection (ESD). PuraStat is applied via a dedicated endoscopic catheter (Top Corporation, Tokyo, Japan) introduced through the working channel of any diagnostic or therapeutic endoscope. PuraStat is comprised of a repeating sequence of amino acid, Arginine (R), Alanine (A), Aspartic Acid (D) and Alanine (A) which form the RADARADARADARADA (RADA16) peptide, which self-assembles into *ß* -sheets. Once the PuraStat gel is in direct contact with blood or body fluid the acidic peptide solution is rapidly neutralised, resulting in the ß-sheets forming a 3-dimensional nano fibre hydrogel scaffold, similar in structure to the extracellular matrix. This hydrogel matrix forms a physical barrier over the bleeding vessel or area to cause haemostasis. The visibility of the bleeding area remains intact and the endoscope view is not obscured due to the transparent nature of the peptide, therefore haemostasis is under direct visual control of the physician. In addition, due to the hydrogel nature of the device the catheter does not become clogged by the product.

After the initial introduction of TDM-621 (PuraStat) for clinical use in post gastric tumour removal in 2014 [6], further reports of the effectiveness of PuraStat came from applications in cardiac and sinus surgery [7, 8]. Currently there is very limited data available investigating clinical experiences with PuraStat in gastrointestinal bleeding. Two recent studies on patients undergoing endoscopic resection showed satisfactory rates of haemostasis and prevention of delayed bleeding, even as compared to cautery, in oesophageal and colonic ESD [9, 10]. Another recent publication presented data from their 3-centre-experience using PuraStat as a rescue therapy for refractory acute gastrointestinal bleeding, following failure of at least 2 standard haemostatic techniques [11].

This prospective, observational pilot study was designed as the first multicentre study to evaluate the feasibility, efficacy and indication profiles of PuraStat in a clinical setting during acute gastrointestinal bleeding.

## Materials and methods

### Patients

Between July 2017 and December 2018 all consecutive patients presenting with gastrointestinal bleeding treated with PuraStat were prospectively enrolled in the study. 15 endoscopy departments were selected for the recruitment of patients. Among these, five centres (listed in the Contributions section) recruited less than 5 patients. Investigators at each participating centre were highly experienced in therapeutic endoscopy. In all centres, a short practical training session on the application of PuraStat was carried out before the first patients were included in the study. Inclusion criteria were age > 18 years, acute upper or lower gastrointestinal bleeding including active bleeding and lesions with signs of recent haemorrhage such as visible vessels, adherent clots or pigmented spots according to the Forrest classification [12] when applicable and a signed consent to participate in the study. Exclusion criterion was the presence of upper variceal bleeding, where the application of PuraStat is not recommended. Established endoscopic therapy techniques prior to the application of PuraStat were permitted, and for analysis patients were divided into subgroups of those who received PuraStat as a first haemostatic treatment option (primary use) or following the failure of established haemostatic techniques (secondary use). As dual therapy is often recommended in guidelines for acute gastrointestinal bleeding, where PuraStat was used as the primary therapy, once haemostasis was achieved, further endoscopic therapy in addition to the application of PuraStat was also permitted and documented, if deemed clinically necessary according to the view of the applying physician and local guidelines.

Hemoglobin (g/dl), platelets (/nl) and international normalised ratio (INR) values before and in the 1–3 days after the procedure were recorded, together with the number of erythrocyte unit transfusions the patient required.

The primary endpoint was the achievement of haemostasis during the procedure with PuraStat (procedure success). This was defined as visual confirmation of haemostasis by the physician following application of PuraStat. The volume of PuraStat used was not defined but was documented, as it is dependent on the level of the bleeding activity. Secondary end points were the prevention of rebleeding (defined as therapy success), the documentation of risk and side effect profiles of PuraStat. Rebleeding was defined as the presence of clinical signs of gastrointestinal bleeding (hematemesis, melena, hematochezia) in association with cardiovascular instability or Hemoglobin drop. A stable clinical condition without signs of rebleeding assessed at 3 and 7 days after the application of PuraStat was defined as therapy success. A routine second-look endoscopy was not required but was documented when performed. The multicentre design in different endoscopic units was chosen to evaluate the feasibility of PuraStat in routine clinical practice.

### Application of PuraStat

**Fig. 1:**
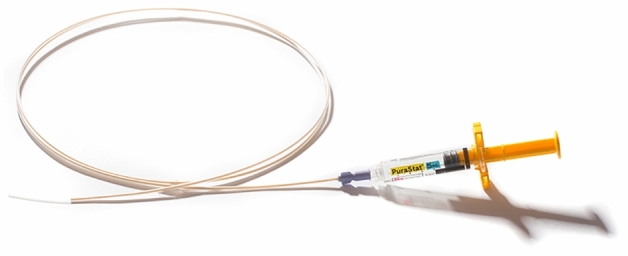
A: PuraStat system ready-to-use. A 5 mL PuraStat syringe is connected with the application catheter (Image by courtesy of 3D Matrix UK, Ltd, London, UK)

The decision to apply PuraStat as a primary or secondary therapy was made with respect to the individual clinical situation of the patient and according to the judgement of the endoscopist. The participating centres decided individually which bleeding lesion or situation might be appropriate in which to utilise PuraStat as a therapy. No specification was made with regards the volume (1 mL, 3 mL or 5 mL) of the gel to be applied (Fig. 1 shows the ready-to-use PuraStat system, 3D Matrix, Europe SAS.).

### Documentation of PuraStat application

After application of PuraStat, the procedural data were documented on a case report form (CRF). The CRF recorded the following data: Localization of the bleeding site, bleeding type according to the Forrest classification, primary or secondary use, previous or additional application of established techniques for haemostasis (submucosal injection of adrenaline or fibrin glue, number of hemoclips, argon plasma coagulation). 3 and 7 days after application, the therapeutic success of PuraStat was documented, defined as a stable clinical condition without signs of rebleeding. If patients had rebleeding, underwent surgery or if another serious adverse event occurred within 30 days following the application of PuraStat, the events were documented separately. All examiners were asked in the CRF about their satisfaction with the use of Purastat. The option was to state yes or no when asked for satisfaction, no specific graduation was requested although free comments on the topic could be reported.

### Ethics

This prospective observational single-arm pilot study was performed in compliance with the Helsinki declaration and approved by the local ethics committee of the Charité—Universitätsmedizin Berlin (Approval No. EA4/082/17). The study was registered at the Germany Registry for Clinical studies (DRKS00012480), additionally all procedures were covered by a special insurance (policy 5701032603017, HDI Gerling Industrieversicherung, 30659 Hannover, Germany) as an essential requirement for the use of PuraStat in a multicentre trial.

### Statistical analysis

The lack of sufficient literature data on the performance of PuraStat in patients with gastrointestinal bleeding did not allow for the calculation of an exact sample size to verify the hypothesis under study conditions. Therefore, a pilot study was designed and an estimation of the number of cases was performed based on current clinical practice in the setting of gastrointestinal bleeding: the enrolment of 97 patients was calculated to achieve an expected haemostasis rate of 90% and a two-sided 95% confidence interval of ± 6%. Descriptive statistics (mean and standard deviation as well as median, range and interquartile ranges) were calculated. Proportions were compared with Chi-Square or Fisher’s exact test. *P*-values were considered significant when < 0.05. The calculation was performed with the software nQuery Version 6.0.

## Results

In this pilot study, 111 patients with gastrointestinal bleeding were included and treated with PuraStat. The baseline characteristics of the patients included (68 men, 43 women) are shown in Table 1. Seventy percent (78/111) of the patients had upper and 30% (33/111) had lower active gastrointestinal bleeding. Peptic ulcer lesions, post-polypectomy bleeding, tumour bleeding were among the most common lesions found.

**Table 1:** Demographic and clinical characteristics of all included study patients

Patients characteristics	
Number of patients	111
Male/Female, *n* (%)	68/43 (61/39)
Age, years (mean ± SD)	M 68 ± 16, F 68 ± 14
Bleeding Site	
**Upper GI-bleeding, *n* (%)**	**78 (70)**
Oesophagus	10 (9)
Stomach	31 (28)
Duodenum/Jejunum	37 (33)
**Lower GI-bleeding, *n* (%)**	**33 (30)**
Right colon	15 (14)
Left colon	18 (16)
Type of lesion, *n* (%)	
Peptic ulcer	42 (38)
Post-polypectomy (EMR/ESD)	28 (25)
Tumour	15 (14)
Vascular lesions (angiectasia)	6 (5)
Colon diverticula	5 (4.5)
Mucosal lesions (e.g. Mallory-Weiss)	2 (2)
Other*	13 (12)
Bleeding Activity, *n* (%)	
Spurting haemorrhage	7 (6)
Oozing haemorrhage	76 (69)
Non-bleeding visible vessel	16 (14)
Adherent clot	6 (5)
Flat pigmented spot	6 (5)

*SD* standard deviation, *DOAC* direct oral anticoagulants, *LMWH* low molecular weight heparin, *ASA* acetylsalicylic acid, *GI* gastrointestinal, *EMR* endoscopic mucosal resection, *ESD* endoscopic submucosal dissection, *PEG* percutaneous endoscopic gastrostomy

*Post-papillotomy, post-biopsy, percutaneous endoscopic gastrostomy (PEG) complication, anastomosis

41 patients (37%) received transfusion of erythrocyte concentrates prior to endoscopy due to hemodynamically relevant bleeding. In 12 patients (11%), impaired blood coagulation or platelet function was observed (INR > 1.5 or thrombocyte counts < 50/nl) at the time of endoscopy, while 15 patients (13.5%) were on anticoagulant or antiplatelet therapy (7 on Acetylsalicylic acid, 5 on low molecular weight heparin (LMWH), 1 on direct oral anticoagulants and 2 on a dual therapy). A total of 12 patients received transfusions of other blood products (platelets, fresh-frozen plasma or prothrombin complex concentrate) and one patient was treated with tranexamic acid before endoscopy.

The bleeding activity was in most cases classified as oozing bleeding (76/111, 69%), in seven cases spurting haemorrhage (6%), in 16 cases a visible vessel (14%) and in 6 cases each (5%) an adherent clot or hematin on the ground of a lesion were observed. In all seven patients with spurting bleeding, PuraStat was applied as a secondary therapy to standard techniques, thus utilising a combination of submucosal injection, hemoclips, over-the-scope clips (OTSC) and gel to reach final haemostasis. After endoscopy therapy, none of these patients required surgery which was an overwhelming positive outcome for these patients.

Analysis of haemostasis rates was performed in the whole cohort as well as in subgroups according to primary or secondary use of PuraStat and site of bleeding (Table 2 and Supplementary Table 1). In 71% of cases (79/111) PuraStat was used as the primary therapy to achieve haemostasis and initial haemostatic success was reached in 74/79 (94%) patients. Within this subgroup, for 43% (32/74) of patients an additional therapeutic tool was applied after initial haemostasis with PuraStat, according to the presentation of the bleeding and when judged necessary by the physician according to local practice and guidelines (in form of submucosal injection, hemoclip, argon plasma coagulation or a combination of these techniques). The therapeutic success rate in the subgroup, where PuraStat was the primary therapy either alone or with the addition of a secondary standard technique, was 91% at 3 days (72/79, 95% CI, 84–96%) and 87% at 7 days (69/79, 95% CI, 80–95%) after the procedure. The rebleeding rate in this subgroup was 18% (14/79). After repeat endoscopy with further treatment (or if repeat endoscopy was not feasible), four patients underwent surgical therapy as a final treatment (5%, 4/79).

**Table 2:** Summary of the outcomes of PuraStat treatment

Outcome of PuraStat treatment	
**Primary use of PuraStat**	***N* = 79**
Procedure success, *n* (%)	74 (94%, 95% CI 88–99)
Application of additional techniques after Purastat*	32/74 (43%, 95% CI 32–54)
**Secondary use of PuraStat**	***N* = 32**
Procedure success, *n* (%)	24 (75%, 95% CI 59–91)
**Outcome**	
Overall procedure success, *n* (%)	98/111 (88%, 95% CI 82–94)
Overall therapeutic success after 3 days, *n* (%)	96/111 (87%, 95% CI 80–93)
Overall therapeutic success after 7 days, *n* (%)	90/111 (81%, 95% CI 72–88)
Rebleeding, *n* (%)	18/111 (16%, 95% CI 10–24)
Within 7 days after the procedure	13
After 7 days	5
With bridging to surgery	5/111

95%*CI* 95% confidence interval

*Additional techniques applied to stabilise haemostasis according to guidelines and/or if deemed necessary by the physician

PuraStat was used in 32/111 (29%) patients as a secondary treatment option after failure of standard techniques, and induced haemostasis in 75% of cases (24/32, 95% CI 59–91%).

The initial procedure success including the whole series was 88% (98/111, 95% CI 82–94%), while the overall therapeutic success rate was 87% (96/111, 95% CI 80–93%) after 3 days and 81% (90/111, 95% CI 72–88) after 7 days. Data on rebleeding rates are shown in Table 2. In 18 out of 111 patients rebleeding was observed and further treatment was necessary: final sustained haemostasis could be achieved in thirteen patients after repeat endoscopy (12%, 13/111), while 5 patients underwent surgery as a definite therapy within 1 week after rebleeding. In those cases, temporary haemostasis with Purastat allowed the stabilisation of the patients, thus possibly playing a role in preventing emergency surgery.

With regard to therapeutic success in patients on anticoagulant/antiplatelet therapy, haemostasis was obtained in 13/15 cases (procedure success 87%, 95% CI 70–100) with no significant differences as compared to patients not taking anticoagulant/antiplatelet agents (*p* = 0.69). Failure of haemostasis achievement was observed in two patients on full-dose LMWH. Rebleeding in this subgroup was observed in 3 cases (one patient taking acetylsalicylic acid and two patients taking LMWH), also with no evidence of significant differences as compared to patients not taking such medications (20% versus 16%, *p* = 0.71). It has to be stressed that the small size of these subgroups likely limits the reliability of this analysis. No therapeutic failure or rebleeding was observed on patients with coagulopathy or thrombocytopenia, who were treated with blood products according to local protocols.

**Fig. 2:**
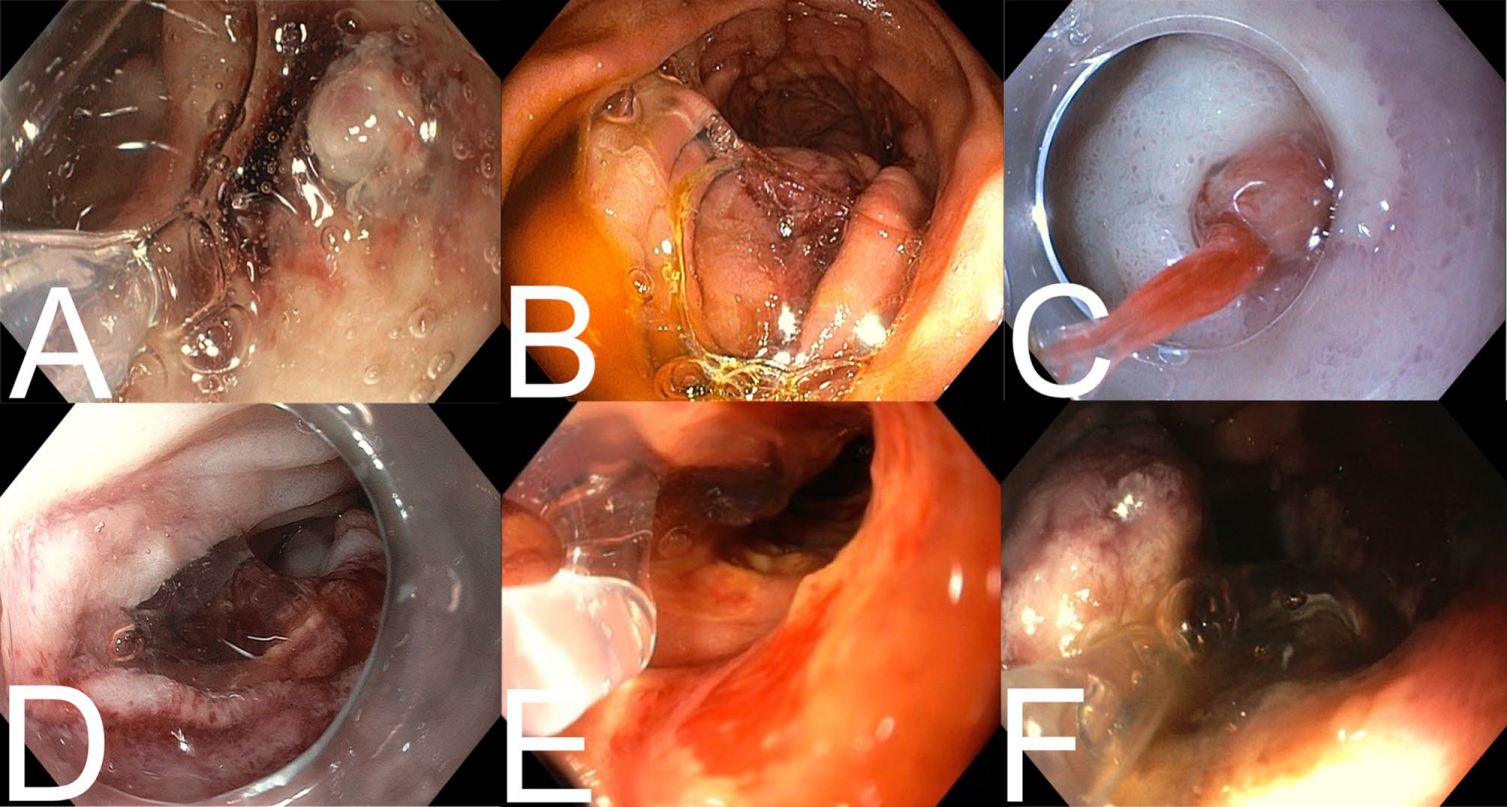
Clinical application of PuraStat in different indications. **A** Duodenal ulcer with vessel and adherent clot. **B** Gastrointestinal stromal tumour in the proximal jejunum after oozing haemorrhage. **C** Duodenal ulcer with visible vessel, stabilisation of the endoscope with distal cap. **D** Oozing bleeding after polypectomy in the duodenum. **E** Tumour bleeding after duodenal infiltration of a pancreas tumour. **F** Duodenal ulcer with vessel and adherent clot

No adverse events due to application of PuraStat or technical failures were reported. The volume of gel required to achieve haemostasis was 3 mL in 59% (65/111), 1 mL in 28% (31/111), 5 mL in 6% (7/111) and a combination (2 mL, 4 mL or 6 mL) in 7% (8/111) of the procedures. Representative images from selected cases are depicted in Fig. 2. The application of PuraStat was considered safe and feasible by all investigators: in 86% of cases (95/111) satisfaction with PuraStat was reported in the CRF. In two cases the operators reported some difficulties due to latency between the start of application and the actual spill of the gel from the tip of the catheter.

## Discussion

The data presented in this observational pilot study found PuraStat to be a promising tool to achieve and help sustain haemostasis in upper and lower gastrointestinal bleeding. To our knowledge, this is the first prospective study with a multicentre design to investigate feasibility, safety and efficacy of PuraStat application for treatment of acute gastrointestinal bleeding in routine clinical practice. We applied PuraStat as a primary treatment, either alone or, following initial haemostatic success, in combination with other established techniques, or as a secondary therapy following failure of established techniques, such as submucosal injection therapy, hemoclips or thermal coagulation.

The largest proportion of patients included in our study were patients with active oozing bleeding, in whom typically a rebleeding rate of up to 50% is reported in the literature [13]; however, in this study we found a rebleeding rate of 12% at 7 days and of 16% at 30 days in our whole patients’ collective. The overall therapeutic success rates in the study were 87% at 3 days and 81% at 7 days including both cases with primary and secondary use of PuraStat.

A large number of technical innovations broaden the field of gastrointestinal bleeding treatment, among these, OTSC [14] and haemostatic powders [4]. This more recent addition to the toolbox for treating Upper and Lower gastrointestinal bleeding is PuraStat. The overall success rate of endoscopic treatment to achieve initial haemostasis with PuraStat observed in our study of 88% (98/111) is comparable or superior to most success rates reported for haemostatic powders. This result is even more relevant considering that our study population included a high prevalence of active bleeding (83/111 (75%) being oozing or spurting haemorrhage). Our group previously reported a treatment success of 64% in upper gastrointestinal bleeding with EndoClot [15], despite reports that revealed a 100% immediate haemostasis success rate in a small series of 21 patients [16]. With regards to Hemospray, after its first trial in 20 patients, [17] a prospective study in 10 European Centres (SEAL survey) examined 63 patients and found a primary haemostasis rate of 76% [18]. Another literature analysis reported a haemostasis success rate of 84.6% in Forrest Ia/b ulcers in thirteen patients [19]. Recent data from Milan, Italy, described successful haemostasis with PuraStat in 91% of the patients [11].

From a clinician’s perspective, PuraStat seems to have some potential advantages over the powders. Notably, the peptide is delivered in a ready-to-use syringe and can be administered immediately via a catheter without any preparation or additional equipment required. Additionally, there is no fear of clotting the application catheter, as can happen with haemostatic powders. However, in contrast to powders PuraStat has to be applied as close as possible to the bleeding point, making direct contact with the tissue.

Both powders and gels are suitable to serve as a bridge to surgery in some patients by stabilising the bleeding situation and hence avoiding immediate emergency surgery [7, 8]. While the effectiveness of PuraStat has been shown in patients who undergo surgery, there are few published studies investigating the role of PuraStat in gastrointestinal bleeding. The available publications, however, do confirm similar haemostatic efficacy results as reported in our study. In one study, the authors described PuraStat to be effective with a success rate of 75% in treating bleeding occurred during endoscopic resection. A very low delayed bleeding rate was found (3%) and the bleeding activity was stopped within 70 s [9]. The application of PuraStat after EMR is feasible and safe which could be shown in 48 patients [20]. The most recent RCT of 101 patients comparing PuraStat to cautery reported a haemostatic efficacy rate of 92.6% for intra procedural oozing bleeds encountered during oesophageal or colonic ESD [10]. In addition, the recent 3-centre-experience paper, concerning PuraStat’s use in acute gastrointestinal bleeding following failure of 2 traditional techniques, reports similar results as found in our data. Initial haemostatic efficacy rate of 90.9% (88% in our data), a rebleed rate of 10.4% within 7 days (12% rebleed rate at 7 days in our data). In the Italian study 27/77 (35%) of the bleeds were non-iatrogenic in origin compared to a larger 70/111 (63%) in our study cohort [11].

As further advantage of PuraStat we report a high degree of satisfaction of the participating centres and operators with the technique, as well as the absence of technical failures and unexpected adverse events.

It has to be noted that this study has potential limitations. The design as a pilot study, without a comparative arm and a randomised treatment assignment, may potentially result in selection bias. Moreover, the possibility of treatment with PuraStat in combination with other well-established endoscopic therapies may make it difficult to distinguish between the efficacy of PuraStat in its own in some cases. However, we find that this study design closely reflects everyday clinical practice when dealing with gastrointestinal bleeding, where guidelines encourage the use of multiple therapeutic tools in combination to achieve haemostasis and where the experience of the physician guides the decision towards the choice of treatment.

In conclusion, PuraStat can be easily and safely applied to control an active gastrointestinal bleeding situation. This biocompatible synthetic peptide is particularly suited as a primary therapeutic option for lesions with oozing type bleeding or lesions with signs of recent haemorrhage. Moreover, PuraStat in combination with other techniques contributes significantly to achieving successful haemostasis in complicated cases of bleeding when used, thus potentially preventing the need for urgent surgical treatment, although this hypothesis requires confirmation with an ad hoc designed trial. Therefore, PuraStat seems to be a useful addition to the therapeutic toolbox for the treatment of acute gastrointestinal bleeding. Further experiences have to be made, especially in moderate or severely inflamed tissue like in the setting of inflammatory bowel disease, where PuraStat might not be adhesive enough to hold the position of application over time.

## Supplementary Information

Below is the link to the electronic supplementary material.

Supplementary file1 (DOCX 16 KB)
