# Supplementary material for: PuraStat in gastrointestinal bleeding: results of a prospective multicentre observational pilot study
Source: Surg Endosc. 2021 Jun 15;36(5):2954–61. doi: 10.1007/s00464-021-08589-6 (PMC9001238; doi:10.1007/s00464-021-08589-6)
Supplement: Supplementary file 1 — Supplementary file1 (DOCX 16 KB) [file 464_2021_8589_MOESM1_ESM.docx]

| **Supplementary Table 1: Stratification of Purastat treatment outcome according to bleeding site, type of lesion and bleeding activity** | | |
| --- | --- | --- |
| **Primary use of PuraStat** | | |
|  | *Procedure success*  *N = 74 (94%)* | *Procedure failure*  *N = 5 (6%)* |
| **Bleeding site** |  |  |
| Esophagus  Stomach  Duodenum  Left Colon  Right Colon  Jejunum/Ileum | 6  14  29  12  12  1 | 2  1  0  2  0  0 |
| **Bleeding lesion** |  |  |
| Ulcer  Tumor  Post EMR/ESD  other | 28  10  18  18 | 2  0  2  1 |
| **Bleeding activity** |  |  |
| Spurting haemorrhage (eq. Ia)  Oozing haemorrhage (eq. Ib)  Visible vessel (eq. IIa)  Adherent clot (eq. IIb)  Flat pigmented spot (eq. IIc) | 3  48  13  5  5 | 0  4  1  0  0 |
|  | | |
| **Secondary use of PuraStat** | | |
|  | *Procedure success*  *N= 24 (75%)* | *Procedure failure*  *N= 8 (25%)* |
| **Bleeding site** |  |  |
| Esophagus  Stomach  Duodenum  Left Colon  Right Colon  Jejunum/Ileum | 2  13  2  4  3  0 | 0  3  5  0  0  0 |
| **Bleeding lesion** |  |  |
| Ulcer  Tumor  Post EMR/ESD  other | 8  5  4  7 | 4  0  0  4 |
| **Bleeding activity** |  | |
| Spurting haemorrhage (eq. Ia)  Oozing haemorrhage (eq. Ib)  Visible vessel (eq. IIa)  Adherent clot (eq. IIb)  Flat pigmented spot (eq. IIc) | 3  18  1  1  1 | 1  6  1  0  0 |
| Eq., equivalent; EMR, endoscopic mucosal resection; ESD, endoscopic submucosal dissection. | | |
